# Tris(2,2′-bipyridine)­copper(II) penta­cyanido­nitro­soferrate(II) methanol disolvate monohydrate

**DOI:** 10.1107/S1600536813006867

**Published:** 2013-03-16

**Authors:** Julia A. Rusanova, Oksana V. Nesterova, Roman I. Zubatyuk, Olesia V. Kozachuk

**Affiliations:** a Department of Inorganic Chemistry, Taras Shevchenko National University of Kyiv, 64/13 Volodymyrska St, Kyiv 01601, Ukraine; b Institute for Scintillation Materials, "Institute for Single Crystals", National Academy of Sciences of Ukraine, Lenina ave. 60, Kharkov 61001, Ukraine

## Abstract

The title complex [Cu(C_10_H_8_N_2_)_3_][Fe(CN)_5_(NO)]·2CH_3_OH·H_2_O, consists of discrete [Cu(bpy)_3_]^2+^ cations (bpy is 2,2′-bipyridine), [Fe(CN)_5_NO]^2−^ anions and solvent mol­ecules of crystallization (two methanol mol­ecules and one water mol­ecules per asymmetric unit). The Cu^II^ ion adopts a distorted octa­hedral environment, coordinated by six N atoms from three bpy ligands. The cation charge is balanced by a nitro­prusside counter-anion, which has a slightly distorted octa­hedral coordination geometry. In the crystal, anions and solvent mol­ecules are involved in O—H⋯N and O—H⋯O hydrogen bonds, which form chains along [100]. The cations are located between these chains.

## Related literature

For background to the direct synthesis of coordination compounds, see: Buvaylo *et al.* (2005 ▶); Babich *et al.* (1996 ▶); Kovbasyuk *et al.* (1998 ▶); Makhankova *et al.* (2002 ▶); Nesterov *et al.* (2006 ▶); Pryma *et al.* (2003 ▶); Vinogradova *et al.* (2002 ▶). For the structures of related complexes, see: Nikitina *et al.* (2008 ▶); Vreshch *et al.* (2009*a* ▶,*b* ▶); Shyu *et al.* (1997 ▶); Shyu & Wei (1999 ▶); Dong *et al.* (2003 ▶); Wang *et al.* (2007 ▶); Zhang *et al.* (2004 ▶).
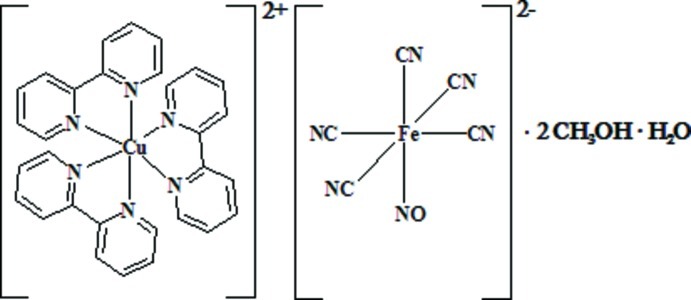


## Experimental

### 

#### Crystal data

[Cu(C_10_H_8_N_2_)_3_][Fe(CN)_5_(NO)]·2CH_4_O·H_2_O
*M* *_r_* = 830.15
Monoclinic, 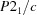

*a* = 11.1308 (8) Å
*b* = 14.7928 (9) Å
*c* = 23.1448 (17) Å
β = 90.916 (8)°
*V* = 3810.4 (5) Å^3^
*Z* = 4
Mo *K*α radiation
μ = 1.00 mm^−1^
*T* = 293 K
0.30 × 0.20 × 0.10 mm

#### Data collection

Oxford Diffraction Xcalibur Sapphire3 diffractometer
Absorption correction: numerical (*CrysAlis PRO*; Oxford Diffraction, 2010 ▶) *T*_min_ = 0.74, *T*_max_ = 0.91
22593 measured reflections
7368 independent reflections
3799 reflections with *I* > 2σ(*I*)
*R*_int_ = 0.053

#### Refinement

*R*[*F* ^2^ > 2σ(*F* ^2^)] = 0.051
*wR*(*F* ^2^) = 0.110
*S* = 1.01
7368 reflections
496 parameters
H-atom parameters constrained
Δρ_max_ = 0.59 e Å^−3^
Δρ_min_ = −0.63 e Å^−3^

### 

Data collection: *CrysAlis PRO* (Oxford Diffraction, 2010 ▶); cell refinement: *CrysAlis PRO*; data reduction: *CrysAlis PRO*; program(s) used to solve structure: *SHELXTL* (Sheldrick, 2008 ▶); program(s) used to refine structure: *SHELXTL*; molecular graphics: *SHELXTL*; software used to prepare material for publication: *publCIF* (Westrip, 2010 ▶).

## Supplementary Material

Crystal structure: contains datablock(s) I, global. DOI: 10.1107/S1600536813006867/lh5592sup1.cif

Structure factors: contains datablock(s) I. DOI: 10.1107/S1600536813006867/lh5592Isup2.hkl

Additional supplementary materials:  crystallographic information; 3D view; checkCIF report

## Figures and Tables

**Table 1:** Hydrogen-bond geometry (Å, °)

*D*—H⋯*A*	*D*—H	H⋯*A*	*D*⋯*A*	*D*—H⋯*A*
O3*S*—H3*SB*⋯N9	0.85	2.04	2.870 (5)	165
O3*S*—H3*SA*⋯N7^i^	0.85	2.25	3.058 (5)	158
O1*S*—H1*S*⋯N8^ii^	0.82	2.08	2.831 (5)	151
O2*S*—H2*S*⋯O3*S* ^iii^	0.82	1.96	2.746 (7)	161

Symmetry codes: (i) 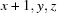; (ii) 
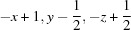
; (iii) 
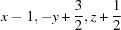
.

**Fig. 1.**
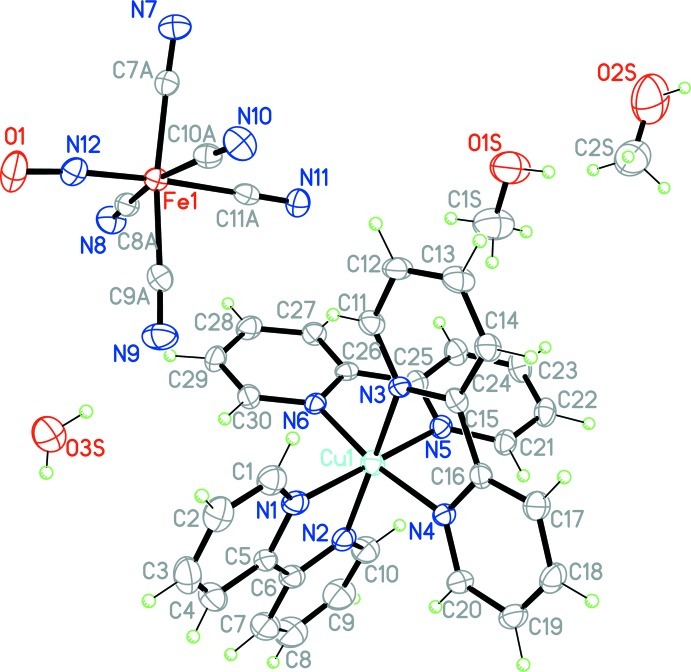
The molecular structure of the title compound. Displacement ellipsoids are drawn at the 50% probability level.

**Fig. 2.**
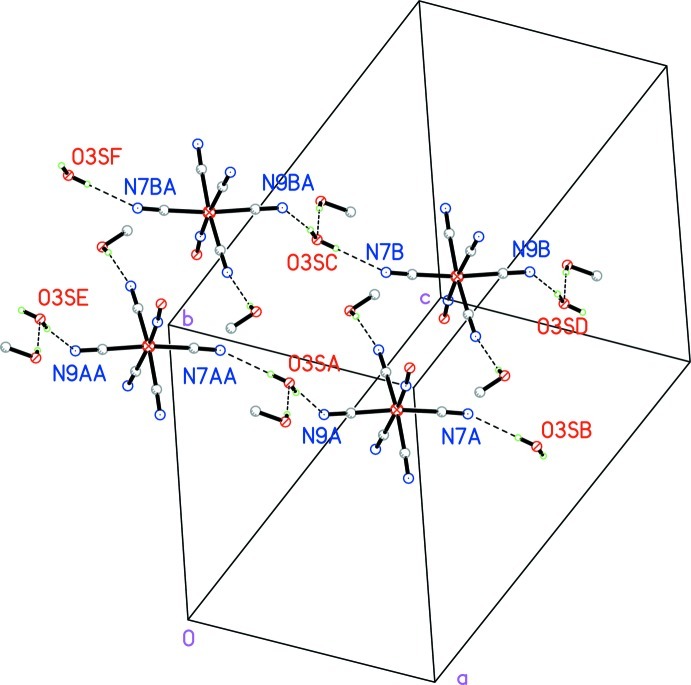
Part of the crystal structure of the title compound with cations omitted for clarity to show the one-dimensional hydrogen-bonded (dashed lines) chains formed by the anions and solvent molecules.

## supplementary crystallographic information

### Comment

This work is a continuation of our research in the field of direct synthesis of coordination compounds (Buvaylo *et al.*, 2005; Babich *et al.*, 1996; Kovbasyuk *et al.*, 1998; Makhankova *et al.*, 2002; Nesterov *et al.*, 2006; Pryma *et al.*, 2003; Vinogradova *et al.*, 2002). We have shown recently the possibility of using anionic complexes as a source of metalloligands in direct synthesis of heterometallic compounds (Nikitina *et al.*, 2008; Vreshch *et al.*, 2009*a*,*b*).

In this paper we present a novel Cu/Fe heterometallic ionic complex [Cu(bpy)_3_][Fe(CN)_5_NO]^.^2CH_3_OH^.^H_2_O which consists of discrete [Cu(bpy)_3_]^2+^ and [Fe(CN)_5_NO]^2-^ ions (Fig. 1). The Cu^II^ ion adopts a distorted octahedral environment by coordinating with six nitrogen atoms from three bpy ligands. The range of Cu—N bond distances of 1.999 (3) - 2.035 (3)Å is in good agreement with the previously reported values for analagous complexes (Shyu *et al.*, 1999; Wang *et al.*, 2007). The anion geometry is unremarkable and in good agreement with reported values for other nitroprussides (Shyu *et al.* 1997; 1999; Dong *et al.* 2003; Zhang *et al.*, 2004). In the crystal, anions are involved in the formation of O—H···O hydrogen bonds with solvent molecules to form one-dimensional chains along [100] (Fig. 2). The complex cations are located between these chains.

### Experimental

Copper powder (0.04 g, 0.63 mmol), NH_4_HSO_4_ (0.145 g, 1.26 mmol), Na_2_[Fe(CN)_5_(NO)]^.^2H_2_O (0.188 g, 0.63 mmol) and bpy (0.296 g, 1.89 mmol) in methanol (30 ml) were heated to 323-333K and stirred magnetically until total dissolution of copper was observed (30 min). Dark-red crystals suitable for X-ray crystallography was isolated from the resulting dark-red solution with addition of 2-propanol in six days. The crystals (0.1 g, yield 30%) were filtered off, washed with dry methanol, and finally dried *in vacuo* at room temperature.

### Refinement

H atoms were included in calculated positions with C—H = 0.93 - 0.96Å and O—H = 0.82 - 0.85Å. They were included in the refinement in a riding-motion approximation with U_iso_(H) = 1.2U_eq_(C) or U_iso_(H) = 1.5U_eq_(O,C_methyl_).

### Crystal data

**Table:**

[Cu(C_10_H_8_N_2_)_3_][Fe(CN)_5_(NO)]·2CH_4_O·H_2_O	*F*(000) = 1708
*M**_r_* = 830.15	*D*_x_ = 1.447 Mg m^−^^3^
Monoclinic, *P*2_1_/*c*	Mo *K*α radiation, λ = 0.71073 Å
Hall symbol: -P 2ybc	Cell parameters from 5205 reflections
*a* = 11.1308 (8) Å	θ = 2.6–32.2°
*b* = 14.7928 (9) Å	µ = 1.00 mm^−^^1^
*c* = 23.1448 (17) Å	*T* = 293 K
β = 90.916 (8)°	Block, dark red
*V* = 3810.4 (5) Å^3^	0.30 × 0.20 × 0.10 mm
*Z* = 4	

### Data collection

**Table:**

Oxford Diffraction Xcalibur Sapphire3 diffractometer	7368 independent reflections
Radiation source: fine-focus sealed tube	3799 reflections with *I* > 2σ(*I*)
Graphite monochromator	*R*_int_ = 0.053
ω scans	θ_max_ = 26.4°, θ_min_ = 2.8°
Absorption correction: numerical (*CrysAlis PRO*; Oxford Diffraction, 2010)	*h* = −13→11
*T*_min_ = 0.74, *T*_max_ = 0.91	*k* = −18→18
22593 measured reflections	*l* = −28→24

### Refinement

**Table:**

Refinement on *F*^2^	Primary atom site location: structure-invariant direct methods
Least-squares matrix: full	Secondary atom site location: difference Fourier map
*R*[*F*^2^ > 2σ(*F*^2^)] = 0.051	Hydrogen site location: inferred from neighbouring sites
*wR*(*F*^2^) = 0.110	H-atom parameters constrained
*S* = 1.01	*w* = 1/[σ^2^(*F*_o_^2^) + (0.042*P*)^2^] where *P* = (*F*_o_^2^ + 2*F*_c_^2^)/3
7368 reflections	(Δ/σ)_max_ = 0.001
496 parameters	Δρ_max_ = 0.59 e Å^−^^3^
0 restraints	Δρ_min_ = −0.63 e Å^−^^3^

### Special details

**Table:**

Geometry. All e.s.d.'s (except the e.s.d. in the dihedral angle between two l.s. planes) are estimated using the full covariance matrix. The cell e.s.d.'s are taken into account individually in the estimation of e.s.d.'s in distances, angles and torsion angles; correlations between e.s.d.'s in cell parameters are only used when they are defined by crystal symmetry. An approximate (isotropic) treatment of cell e.s.d.'s is used for estimating e.s.d.'s involving l.s. planes.
Refinement. Refinement of *F*^2^ against ALL reflections. The weighted *R*-factor *wR* and goodness of fit *S* are based on *F*^2^, conventional *R*-factors *R* are based on *F*, with *F* set to zero for negative *F*^2^. The threshold expression of *F*^2^ > σ(*F*^2^) is used only for calculating *R*-factors(gt) *etc*. and is not relevant to the choice of reflections for refinement. *R*-factors based on *F*^2^ are statistically about twice as large as those based on *F*, and *R*- factors based on ALL data will be even larger.

### Fractional atomic coordinates and isotropic or equivalent isotropic displacement parameters (Å^2^)

**Table:**

	*x*	*y*	*z*	*U*_iso_*/*U*_eq_	
Fe1	0.54777 (5)	0.85655 (4)	0.08468 (3)	0.03893 (16)	
Cu1	0.91414 (4)	0.80913 (3)	0.32784 (2)	0.04640 (16)	
N1	1.0220 (3)	0.7802 (2)	0.26108 (16)	0.0497 (9)	
N2	1.0506 (3)	0.8936 (2)	0.34517 (16)	0.0495 (9)	
N3	0.7880 (3)	0.7182 (2)	0.30682 (14)	0.0382 (8)	
N4	0.9782 (3)	0.7012 (2)	0.37086 (13)	0.0378 (8)	
N5	0.8102 (3)	0.8510 (2)	0.39425 (14)	0.0416 (8)	
N6	0.8275 (3)	0.9133 (2)	0.29055 (14)	0.0372 (8)	
N7	0.2746 (3)	0.8839 (2)	0.06854 (17)	0.0620 (11)	
N8	0.5926 (3)	1.0615 (2)	0.09581 (18)	0.0622 (11)	
N9	0.8113 (4)	0.8198 (3)	0.11772 (19)	0.0731 (12)	
N10	0.5011 (3)	0.6536 (3)	0.10134 (18)	0.0637 (11)	
N11	0.4997 (3)	0.8746 (2)	0.21488 (17)	0.0534 (10)	
N12	0.5814 (3)	0.8554 (2)	0.01612 (17)	0.0492 (9)	
C1	1.0021 (4)	0.7186 (3)	0.2195 (2)	0.0605 (12)	
H1A	0.9292	0.6879	0.2192	0.073*	
C2	1.0812 (5)	0.6988 (3)	0.1786 (2)	0.0694 (14)	
H2A	1.0634	0.6554	0.1507	0.083*	
C3	1.1885 (5)	0.7432 (4)	0.1783 (2)	0.0765 (16)	
H3A	1.2449	0.7309	0.1501	0.092*	
C4	1.2113 (4)	0.8055 (4)	0.2197 (2)	0.0739 (15)	
H4A	1.2843	0.8361	0.2203	0.089*	
C5	1.1273 (4)	0.8241 (3)	0.2611 (2)	0.0502 (11)	
C6	1.1399 (4)	0.8914 (3)	0.3066 (2)	0.0551 (12)	
C7A	0.3757 (4)	0.8748 (3)	0.07377 (18)	0.0445 (10)	
C7	1.2363 (5)	0.9509 (4)	0.3111 (3)	0.0795 (16)	
H7A	1.3002	0.9470	0.2858	0.095*	
C8A	0.5745 (3)	0.9855 (3)	0.09130 (19)	0.0474 (11)	
C8	1.2348 (5)	1.0162 (4)	0.3542 (3)	0.0928 (19)	
H8A	1.2970	1.0580	0.3572	0.111*	
C9A	0.7126 (4)	0.8341 (3)	0.10677 (19)	0.0474 (11)	
C9	1.1436 (5)	1.0192 (4)	0.3915 (3)	0.0841 (17)	
H9A	1.1416	1.0631	0.4203	0.101*	
C10A	0.5162 (3)	0.7285 (3)	0.09489 (19)	0.0456 (11)	
C10	1.0541 (4)	0.9564 (3)	0.3865 (2)	0.0634 (13)	
H10A	0.9925	0.9576	0.4131	0.076*	
C11A	0.5175 (3)	0.8666 (3)	0.1664 (2)	0.0415 (10)	
C11	0.6953 (4)	0.7304 (3)	0.2706 (2)	0.0497 (11)	
H11A	0.6908	0.7842	0.2499	0.060*	
C12	0.6073 (4)	0.6684 (3)	0.2625 (2)	0.0546 (12)	
H12A	0.5442	0.6794	0.2367	0.066*	
C13	0.6126 (4)	0.5887 (3)	0.2931 (2)	0.0586 (13)	
H13A	0.5521	0.5457	0.2891	0.070*	
C14	0.7075 (4)	0.5742 (3)	0.32919 (19)	0.0499 (11)	
H14A	0.7130	0.5207	0.3501	0.060*	
C15	0.7955 (3)	0.6383 (3)	0.33483 (17)	0.0409 (10)	
C16	0.9060 (4)	0.6278 (3)	0.37029 (17)	0.0413 (10)	
C17	0.9377 (4)	0.5503 (3)	0.39901 (19)	0.0548 (12)	
H17A	0.8866	0.5005	0.3985	0.066*	
C18	1.0445 (4)	0.5465 (3)	0.4283 (2)	0.0587 (13)	
H18A	1.0673	0.4940	0.4477	0.070*	
C19	1.1185 (4)	0.6208 (3)	0.42895 (19)	0.0575 (12)	
H19A	1.1918	0.6197	0.4487	0.069*	
C20	1.0814 (4)	0.6969 (3)	0.39965 (18)	0.0476 (11)	
H20A	1.1311	0.7475	0.4001	0.057*	
C21	0.8078 (4)	0.8163 (3)	0.44769 (18)	0.0479 (11)	
H21A	0.8631	0.7716	0.4577	0.058*	
C22	0.7276 (4)	0.8438 (3)	0.48790 (19)	0.0544 (12)	
H22A	0.7277	0.8176	0.5244	0.065*	
C23	0.6471 (4)	0.9102 (3)	0.4740 (2)	0.0600 (12)	
H23A	0.5912	0.9294	0.5008	0.072*	
C24	0.6497 (4)	0.9482 (3)	0.41990 (19)	0.0535 (12)	
H24A	0.5957	0.9937	0.4097	0.064*	
C25	0.7321 (3)	0.9188 (2)	0.38118 (18)	0.0399 (10)	
C26	0.7462 (3)	0.9554 (2)	0.32274 (18)	0.0383 (9)	
C27	0.6835 (3)	1.0305 (3)	0.30261 (19)	0.0479 (11)	
H27A	0.6281	1.0595	0.3259	0.058*	
C28	0.7047 (4)	1.0611 (3)	0.2478 (2)	0.0556 (12)	
H28A	0.6630	1.1108	0.2333	0.067*	
C29	0.7865 (4)	1.0186 (3)	0.21512 (19)	0.0535 (11)	
H29A	0.8024	1.0394	0.1781	0.064*	
C30	0.8463 (4)	0.9441 (3)	0.23692 (18)	0.0472 (11)	
H30A	0.9012	0.9143	0.2138	0.057*	
O1	0.6132 (3)	0.8586 (3)	−0.03055 (15)	0.0856 (11)	
O1S	0.3532 (3)	0.7337 (3)	0.3573 (2)	0.1165 (16)	
H1S	0.3516	0.6904	0.3794	0.175*	
C1S	0.4416 (5)	0.7904 (4)	0.3736 (3)	0.105 (2)	
H1SA	0.4424	0.8416	0.3482	0.158*	
H1SB	0.5173	0.7595	0.3720	0.158*	
H1SC	0.4282	0.8104	0.4125	0.158*	
O2S	0.0815 (7)	0.8683 (4)	0.5202 (3)	0.178 (2)	
H2S	0.0561	0.8220	0.5353	0.267*	
C2S	0.2053 (8)	0.8635 (5)	0.5160 (4)	0.156 (4)	
H2SA	0.2349	0.9175	0.4982	0.234*	
H2SB	0.2262	0.8121	0.4930	0.234*	
H2SC	0.2405	0.8573	0.5540	0.234*	
O3S	1.0227 (3)	0.8062 (2)	0.04880 (15)	0.0899 (11)	
H3SA	1.0827	0.8365	0.0613	0.135*	
H3SB	0.9690	0.8129	0.0742	0.135*	

### Atomic displacement parameters (Å^2^)

**Table:**

	*U*^11^	*U*^22^	*U*^33^	*U*^12^	*U*^13^	*U*^23^
Fe1	0.0380 (3)	0.0344 (3)	0.0444 (3)	0.0019 (3)	0.0002 (3)	0.0001 (3)
Cu1	0.0481 (3)	0.0407 (3)	0.0504 (3)	−0.0027 (2)	0.0019 (3)	0.0039 (3)
N1	0.045 (2)	0.045 (2)	0.059 (2)	0.0040 (17)	−0.006 (2)	0.008 (2)
N2	0.049 (2)	0.046 (2)	0.054 (2)	−0.0050 (18)	−0.011 (2)	0.014 (2)
N3	0.0303 (18)	0.0397 (18)	0.045 (2)	0.0001 (15)	−0.0026 (17)	0.0103 (17)
N4	0.0359 (19)	0.0405 (18)	0.0371 (18)	0.0027 (15)	−0.0009 (17)	0.0014 (17)
N5	0.044 (2)	0.0390 (18)	0.041 (2)	−0.0027 (16)	−0.0012 (17)	0.0017 (18)
N6	0.0386 (19)	0.0361 (17)	0.0368 (19)	−0.0056 (15)	−0.0055 (17)	0.0013 (17)
N7	0.047 (2)	0.065 (2)	0.073 (3)	0.006 (2)	−0.008 (2)	−0.007 (2)
N8	0.064 (2)	0.041 (2)	0.081 (3)	−0.0053 (19)	−0.002 (2)	0.005 (2)
N9	0.048 (2)	0.091 (3)	0.081 (3)	0.015 (2)	−0.003 (2)	−0.016 (3)
N10	0.070 (3)	0.041 (2)	0.080 (3)	−0.004 (2)	0.009 (2)	−0.004 (2)
N11	0.061 (2)	0.051 (2)	0.049 (2)	0.0053 (18)	0.004 (2)	0.005 (2)
N12	0.055 (2)	0.0411 (19)	0.051 (2)	0.0139 (18)	−0.0027 (19)	0.004 (2)
C1	0.062 (3)	0.060 (3)	0.060 (3)	0.009 (3)	0.000 (3)	0.002 (3)
C2	0.086 (4)	0.063 (3)	0.060 (3)	0.010 (3)	0.002 (3)	0.000 (3)
C3	0.089 (4)	0.077 (4)	0.065 (4)	0.017 (3)	0.023 (3)	0.013 (3)
C4	0.053 (3)	0.084 (4)	0.085 (4)	−0.006 (3)	0.009 (3)	0.028 (4)
C5	0.035 (2)	0.059 (3)	0.057 (3)	0.001 (2)	−0.001 (2)	0.021 (3)
C6	0.044 (3)	0.052 (3)	0.069 (3)	−0.011 (2)	−0.010 (3)	0.019 (3)
C7A	0.051 (3)	0.040 (2)	0.042 (2)	0.000 (2)	0.000 (2)	−0.003 (2)
C7	0.062 (3)	0.087 (4)	0.089 (4)	−0.029 (3)	−0.011 (3)	0.020 (4)
C8A	0.042 (2)	0.046 (3)	0.054 (3)	0.000 (2)	−0.001 (2)	0.009 (2)
C8	0.084 (4)	0.078 (4)	0.116 (5)	−0.043 (4)	−0.024 (4)	0.016 (4)
C9A	0.051 (3)	0.042 (2)	0.049 (3)	0.000 (2)	0.009 (2)	−0.006 (2)
C9	0.092 (4)	0.068 (3)	0.092 (4)	−0.022 (3)	−0.020 (4)	−0.003 (3)
C10A	0.039 (2)	0.045 (2)	0.053 (3)	0.006 (2)	0.001 (2)	−0.007 (2)
C10	0.065 (3)	0.054 (3)	0.071 (3)	−0.014 (3)	−0.009 (3)	0.001 (3)
C11A	0.031 (2)	0.030 (2)	0.063 (3)	0.0012 (17)	−0.001 (2)	0.004 (2)
C11	0.043 (3)	0.041 (2)	0.065 (3)	−0.004 (2)	0.002 (3)	0.009 (2)
C12	0.039 (2)	0.054 (3)	0.070 (3)	−0.006 (2)	−0.012 (2)	0.005 (3)
C13	0.051 (3)	0.044 (3)	0.080 (4)	−0.014 (2)	−0.006 (3)	−0.005 (3)
C14	0.051 (3)	0.038 (2)	0.060 (3)	−0.004 (2)	0.001 (3)	0.008 (2)
C15	0.041 (2)	0.037 (2)	0.046 (2)	−0.002 (2)	0.008 (2)	0.002 (2)
C16	0.043 (2)	0.040 (2)	0.041 (2)	0.0016 (19)	0.006 (2)	0.005 (2)
C17	0.058 (3)	0.041 (2)	0.065 (3)	−0.002 (2)	−0.001 (3)	0.012 (2)
C18	0.066 (3)	0.054 (3)	0.057 (3)	0.019 (3)	0.001 (3)	0.014 (3)
C19	0.045 (3)	0.080 (3)	0.047 (3)	0.018 (3)	−0.001 (2)	0.000 (3)
C20	0.042 (3)	0.050 (3)	0.050 (3)	0.002 (2)	−0.006 (2)	0.001 (2)
C21	0.055 (3)	0.043 (2)	0.045 (3)	−0.005 (2)	−0.003 (2)	0.006 (2)
C22	0.075 (3)	0.045 (3)	0.043 (3)	−0.015 (2)	−0.002 (3)	0.003 (2)
C23	0.077 (3)	0.052 (3)	0.052 (3)	−0.002 (3)	0.021 (3)	−0.006 (3)
C24	0.059 (3)	0.043 (2)	0.059 (3)	0.004 (2)	0.009 (3)	0.004 (2)
C25	0.044 (2)	0.033 (2)	0.044 (2)	−0.0062 (19)	0.003 (2)	−0.002 (2)
C26	0.033 (2)	0.035 (2)	0.046 (2)	−0.0043 (18)	−0.008 (2)	−0.002 (2)
C27	0.044 (2)	0.043 (2)	0.057 (3)	0.007 (2)	0.003 (2)	0.002 (2)
C28	0.054 (3)	0.046 (3)	0.066 (3)	0.005 (2)	−0.010 (3)	0.013 (3)
C29	0.060 (3)	0.053 (3)	0.047 (3)	−0.004 (2)	−0.005 (2)	0.017 (2)
C30	0.047 (3)	0.050 (3)	0.044 (3)	−0.005 (2)	0.003 (2)	0.002 (2)
O1	0.110 (3)	0.098 (3)	0.050 (2)	0.035 (2)	0.013 (2)	0.014 (2)
O1S	0.088 (3)	0.082 (3)	0.179 (5)	−0.010 (2)	−0.029 (3)	0.043 (3)
C1S	0.088 (4)	0.071 (4)	0.156 (6)	−0.005 (3)	−0.022 (4)	0.000 (4)
O2S	0.218 (6)	0.121 (4)	0.195 (6)	0.030 (5)	0.014 (6)	0.055 (4)
C2S	0.176 (8)	0.128 (6)	0.161 (8)	−0.077 (7)	−0.073 (7)	0.047 (6)
O3S	0.079 (2)	0.111 (3)	0.081 (3)	−0.002 (2)	0.013 (2)	−0.002 (2)

### Geometric parameters (Å, º)

**Table:**

Fe1—N12	1.636 (4)	C11—C12	1.353 (5)
Fe1—C9A	1.926 (5)	C11—H11A	0.9300
Fe1—C11A	1.932 (5)	C12—C13	1.376 (6)
Fe1—C8A	1.936 (4)	C12—H12A	0.9300
Fe1—C10A	1.942 (5)	C13—C14	1.353 (6)
Fe1—C7A	1.947 (5)	C13—H13A	0.9300
Cu1—N3	1.999 (3)	C14—C15	1.368 (5)
Cu1—N2	2.002 (3)	C14—H14A	0.9300
Cu1—N6	2.006 (3)	C15—C16	1.476 (5)
Cu1—N4	2.006 (3)	C16—C17	1.370 (5)
Cu1—N1	2.018 (4)	C17—C18	1.360 (6)
Cu1—N5	2.035 (3)	C17—H17A	0.9300
N1—C5	1.340 (5)	C18—C19	1.374 (6)
N1—C1	1.341 (5)	C18—H18A	0.9300
N2—C10	1.333 (5)	C19—C20	1.374 (6)
N2—C6	1.347 (5)	C19—H19A	0.9300
N3—C11	1.331 (5)	C20—H20A	0.9300
N3—C15	1.349 (5)	C21—C22	1.362 (6)
N4—C20	1.321 (5)	C21—H21A	0.9300
N4—C16	1.350 (5)	C22—C23	1.364 (6)
N5—C21	1.340 (5)	C22—H22A	0.9300
N5—C25	1.359 (5)	C23—C24	1.374 (6)
N6—C26	1.336 (5)	C23—H23A	0.9300
N6—C30	1.342 (5)	C24—C25	1.364 (5)
N7—C7A	1.138 (5)	C24—H24A	0.9300
N8—C8A	1.147 (5)	C25—C26	1.467 (5)
N9—C9A	1.144 (5)	C26—C27	1.389 (5)
N10—C10A	1.130 (5)	C27—C28	1.371 (6)
N11—C11A	1.149 (5)	C27—H27A	0.9300
N12—O1	1.143 (4)	C28—C29	1.349 (6)
C1—C2	1.337 (6)	C28—H28A	0.9300
C1—H1A	0.9300	C29—C30	1.379 (5)
C2—C3	1.363 (7)	C29—H29A	0.9300
C2—H2A	0.9300	C30—H30A	0.9300
C3—C4	1.351 (7)	O1S—C1S	1.342 (6)
C3—H3A	0.9300	O1S—H1S	0.8200
C4—C5	1.377 (6)	C1S—H1SA	0.9600
C4—H4A	0.9300	C1S—H1SB	0.9600
C5—C6	1.455 (6)	C1S—H1SC	0.9602
C6—C7	1.390 (6)	O2S—C2S	1.384 (8)
C7—C8	1.389 (8)	O2S—H2S	0.8201
C7—H7A	0.9300	C2S—H2SA	0.9599
C8—C9	1.345 (8)	C2S—H2SB	0.9601
C8—H8A	0.9300	C2S—H2SC	0.9601
C9—C10	1.365 (6)	O3S—H3SA	0.8500
C9—H9A	0.9300	O3S—H3SB	0.8499
C10—H10A	0.9300		
			
N12—Fe1—C9A	91.36 (17)	N2—C10—C9	123.3 (5)
N12—Fe1—C11A	175.03 (17)	N2—C10—H10A	118.4
C9A—Fe1—C11A	86.19 (17)	C9—C10—H10A	118.4
N12—Fe1—C8A	92.83 (18)	N11—C11A—Fe1	178.5 (4)
C9A—Fe1—C8A	90.23 (16)	N3—C11—C12	123.1 (4)
C11A—Fe1—C8A	82.87 (17)	N3—C11—H11A	118.4
N12—Fe1—C10A	98.79 (18)	C12—C11—H11A	118.4
C9A—Fe1—C10A	88.45 (16)	C11—C12—C13	119.0 (4)
C11A—Fe1—C10A	85.48 (17)	C11—C12—H12A	120.5
C8A—Fe1—C10A	168.33 (19)	C13—C12—H12A	120.5
N12—Fe1—C7A	96.65 (17)	C14—C13—C12	118.6 (4)
C9A—Fe1—C7A	171.75 (17)	C14—C13—H13A	120.7
C11A—Fe1—C7A	85.97 (16)	C12—C13—H13A	120.7
C8A—Fe1—C7A	91.33 (16)	C13—C14—C15	120.0 (4)
C10A—Fe1—C7A	88.39 (16)	C13—C14—H14A	120.0
N3—Cu1—N2	175.02 (14)	C15—C14—H14A	120.0
N3—Cu1—N6	94.69 (12)	N3—C15—C14	121.5 (4)
N2—Cu1—N6	87.97 (12)	N3—C15—C16	113.8 (3)
N3—Cu1—N4	80.07 (13)	C14—C15—C16	124.7 (4)
N2—Cu1—N4	97.75 (13)	N4—C16—C17	121.4 (4)
N6—Cu1—N4	171.67 (13)	N4—C16—C15	114.3 (3)
N3—Cu1—N1	95.46 (13)	C17—C16—C15	124.3 (4)
N2—Cu1—N1	80.02 (15)	C18—C17—C16	119.5 (4)
N6—Cu1—N1	96.94 (13)	C18—C17—H17A	120.2
N4—Cu1—N1	90.04 (12)	C16—C17—H17A	120.2
N3—Cu1—N5	89.03 (13)	C17—C18—C19	119.4 (4)
N2—Cu1—N5	95.60 (14)	C17—C18—H18A	120.3
N6—Cu1—N5	79.41 (13)	C19—C18—H18A	120.3
N4—Cu1—N5	93.94 (13)	C18—C19—C20	118.4 (4)
N1—Cu1—N5	174.46 (13)	C18—C19—H19A	120.8
C5—N1—C1	117.7 (4)	C20—C19—H19A	120.8
C5—N1—Cu1	115.4 (3)	N4—C20—C19	122.7 (4)
C1—N1—Cu1	126.8 (3)	N4—C20—H20A	118.6
C10—N2—C6	118.6 (4)	C19—C20—H20A	118.6
C10—N2—Cu1	126.3 (3)	N5—C21—C22	122.6 (4)
C6—N2—Cu1	114.7 (3)	N5—C21—H21A	118.7
C11—N3—C15	117.6 (3)	C22—C21—H21A	118.7
C11—N3—Cu1	126.6 (3)	C21—C22—C23	119.3 (4)
C15—N3—Cu1	115.8 (3)	C21—C22—H22A	120.4
C20—N4—C16	118.6 (3)	C23—C22—H22A	120.4
C20—N4—Cu1	126.0 (3)	C22—C23—C24	119.1 (4)
C16—N4—Cu1	115.4 (3)	C22—C23—H23A	120.5
C21—N5—C25	117.8 (3)	C24—C23—H23A	120.5
C21—N5—Cu1	126.9 (3)	C25—C24—C23	119.5 (4)
C25—N5—Cu1	115.2 (3)	C25—C24—H24A	120.2
C26—N6—C30	118.3 (3)	C23—C24—H24A	120.2
C26—N6—Cu1	116.3 (3)	N5—C25—C24	121.6 (4)
C30—N6—Cu1	125.3 (3)	N5—C25—C26	113.6 (3)
O1—N12—Fe1	174.4 (4)	C24—C25—C26	124.8 (4)
C2—C1—N1	123.6 (5)	N6—C26—C27	121.8 (4)
C2—C1—H1A	118.2	N6—C26—C25	115.2 (3)
N1—C1—H1A	118.2	C27—C26—C25	123.0 (4)
C1—C2—C3	119.1 (5)	C28—C27—C26	118.8 (4)
C1—C2—H2A	120.5	C28—C27—H27A	120.6
C3—C2—H2A	120.5	C26—C27—H27A	120.6
C4—C3—C2	118.7 (5)	C29—C28—C27	119.5 (4)
C4—C3—H3A	120.7	C29—C28—H28A	120.2
C2—C3—H3A	120.7	C27—C28—H28A	120.2
C3—C4—C5	120.5 (5)	C28—C29—C30	119.6 (4)
C3—C4—H4A	119.7	C28—C29—H29A	120.2
C5—C4—H4A	119.7	C30—C29—H29A	120.2
N1—C5—C4	120.5 (5)	N6—C30—C29	121.9 (4)
N1—C5—C6	113.9 (4)	N6—C30—H30A	119.0
C4—C5—C6	125.6 (5)	C29—C30—H30A	119.0
N2—C6—C7	120.9 (5)	C1S—O1S—H1S	109.6
N2—C6—C5	115.6 (4)	O1S—C1S—H1SA	109.6
C7—C6—C5	123.5 (5)	O1S—C1S—H1SB	109.4
N7—C7A—Fe1	178.1 (4)	H1SA—C1S—H1SB	109.5
C8—C7—C6	118.4 (5)	O1S—C1S—H1SC	109.4
C8—C7—H7A	120.8	H1SA—C1S—H1SC	109.5
C6—C7—H7A	120.8	H1SB—C1S—H1SC	109.5
N8—C8A—Fe1	178.6 (4)	C2S—O2S—H2S	109.7
C9—C8—C7	120.2 (5)	O2S—C2S—H2SA	109.7
C9—C8—H8A	119.9	O2S—C2S—H2SB	109.3
C7—C8—H8A	119.9	H2SA—C2S—H2SB	109.5
N9—C9A—Fe1	177.3 (4)	O2S—C2S—H2SC	109.4
C8—C9—C10	118.7 (6)	H2SA—C2S—H2SC	109.5
C8—C9—H9A	120.7	H2SB—C2S—H2SC	109.5
C10—C9—H9A	120.7	H3SA—O3S—H3SB	105.2
N10—C10A—Fe1	178.0 (4)		

### Hydrogen-bond geometry (Å, º)

**Table:**

*D*—H···*A*	*D*—H	H···*A*	*D*···*A*	*D*—H···*A*
O3*S*—H3*SB*···N9	0.85	2.04	2.870 (5)	165
O3*S*—H3*SA*···N7^i^	0.85	2.25	3.058 (5)	158
O1*S*—H1*S*···N8^ii^	0.82	2.08	2.831 (5)	151
O2*S*—H2*S*···O3*S*^iii^	0.82	1.96	2.746 (7)	161

Symmetry codes: (i) *x*+1, *y*, *z*; (ii) −*x*+1, *y*−1/2, −*z*+1/2; (iii) *x*−1, −*y*+3/2, *z*+1/2.

## References

[bb1] Babich, O. A., Kokozay, V. N. & Pavlenko, V. A. (1996). *Polyhedron*, **15**, 2727–2731.

[bb2] Buvaylo, E. A., Kokozay, V. N., Vassilyeva, O. Yu., Skelton, B. W., Jezierska, J., Brunel, L. C. & Ozarowski, A. (2005). *Chem. Commun.* pp. 4976–4978. 10.1039/b509810f 16205819

[bb3] Dong, W., Si, S.-F., Liao, D.-Z., Jiang, Z.-H. & Yan, A.-P. (2003). *J. Coord. Chem.* **56**, 531–538.

[bb4] Kovbasyuk, L. A., Vassilyeva, O. Yu., Kokozay, V. N., Linert, W., Reedijk, J., Skelton, B. W. & Oliver, A. G. (1998). *J* *Chem* *Soc* *Dalton Trans* pp. 2735–2738.

[bb5] Makhankova, V. G., Vassilyeva, O. Yu., Kokozay, V. N., Skelton, B. W., Sorace, L. & Gatteschi, D. (2002). *J. Chem. Soc. Dalton Trans.* pp. 4253–4259.

[bb6] Nesterov, D. S., Kokozay, V. N., Dyakonenko, V. V., Shishkin, O. V., Jezierska, J., Ozarowski, A., Kirillov, A. M., Kopylovich, M. N. & Pombeiro, A. J. L. (2006). *Chem. Commun.* pp. 4605–4607. 10.1039/b608790f 17082857

[bb7] Nikitina, V. M., Nesterova, O. V., Kokozay, V. N., Goreshnik, E. A. & Jezierska, J. (2008). *Polyhedron*, **27**, 2426–2430.

[bb8] Oxford Diffraction (2010). *CrysAlis PRO* Oxford Diffraction Ltd, Yarnton, England.

[bb9] Pryma, O. V., Petrusenko, S. R., Kokozay, V. N., Shishkin, O. V. & Teplytska, T. S. (2003). *Eur. J. Inorg. Chem.* pp. 1426–1432.

[bb10] Sheldrick, G. M. (2008). *Acta Cryst.* A**64**, 112–122. 10.1107/S0108767307043930 18156677

[bb11] Shyu, H. L. & Wei, H. H. (1999). *J. Coord. Chem.* **47**, 319–330.

[bb12] Shyu, H. L., Wei, H. H. & Wang, Y. (1997). *Inorg. Chim. Acta*, **258**, 81–86.

[bb13] Vinogradova, E. A., Vassilyeva, O. Yu., Kokozay, V. N., Skelton, B. W., Bjernemose, J. K. & Raithby, P. R. (2002). *J. Chem. Soc. Dalton Trans.* pp. 4248–4252.

[bb14] Vreshch, O. V., Nesterova, O. V., Kokozay, V. N., Dyakonenko, V. V., Shishkin, O. V., Cormary, B., Malfant, I. & Jezierska, J. (2009*a*). *Z. Anorg. Allg. Chem.* **635**, 2316–2323.

[bb15] Vreshch, O. V., Nesterova, O. V., Kokozay, V. N., Skelton, B. W., Garcia, C. J. G. & Jezierska, J. (2009*b*). *Inorg. Chem. Commun.* **12**, 890–894.

[bb16] Wang, L., Yang, X.-Y. & Huang, W. (2007). *Acta Cryst.* E**63**, m835–m836.

[bb17] Westrip, S. P. (2010). *J. Appl. Cryst.* **43**, 920–925.

[bb18] Zhang, B.-F., Xie, C.-Z., Wang, X.-Q., Shen, G.-Q. & Shen, D.-Z. (2004). *Acta Cryst.* E**60**, m1293–m1295. 10.1107/S0108270104021456 15467136

